# Human CD8+ T Cells in Asthma: Possible Pathways and Roles for NK-Like Subtypes

**DOI:** 10.3389/fimmu.2016.00638

**Published:** 2016-12-23

**Authors:** Olga Lourenço, Ana Mafalda Fonseca, Luis Taborda-Barata

**Affiliations:** ^1^CICS – UBI, Health Sciences Research Centre, University of Beira Interior, Covilhã, Portugal; ^2^Department of Allergy and Clinical Immunology, Cova da Beira Hospital Centre, Covilhã, Portugal

**Keywords:** asthma, CD8, CD28, CD27, CD57, human, NK-like, T cells

## Abstract

Asthma affects approximately 300 million people worldwide and is the most common chronic lung disease, which usually is associated with bronchial inflammation. Most research has focused upon the role of CD4+ T cells, and relatively few studies have addressed the phenotypic and functional roles of CD8+ T cell types and subtypes. Human NK-like CD8+ T cells may involve cells that have been described as CD8+CD28−, CD8+CD28−CD57+, CD8+CD27−, or CD8+ effector memory (TEM) cells, among other. However, most of the data that are available regarding these various cell types were obtained in murine models did not thoroughly characterize these cells with phenotypically or functionally or did not involve asthma-related settings. Nevertheless, one may conceptualize three principal roles for human NK-like CD8+ T cells in asthma: disease-promoting, regulatory, and/or tissue repair. Although evidence for some of these roles is scarce, it is possible to extrapolate some data from overlapping or related CD8+ T cell phenotypes, with caution. Clearly, further research is warranted, namely in terms of thorough functional and phenotypic characterization of human NK-like CD8+ T cells in human asthma of varying severity.

## General Aspects about Asthma

Asthma affects approximately 300 million people worldwide and is the most common chronic lung disease (1). It is defined by a history of respiratory symptoms such as wheeze, shortness of breath, chest tightness, and cough that vary over time and in intensity, together with variable expiratory airflow limitation (2). In addition, it is a heterogeneous disease, usually characterized by chronic airway inflammation (2).

## General Aspects about the Role of CD8+ T Cells in Human Asthma

Although there are different clinical and cellular phenotypes in asthma, most research in terms of the role of T lymphocytes in this disease has been focused upon CD4+ T cells in the context of chronic airway inflammation. These CD4+ T cells produce various cytokines, namely IL-4, IL-9, and IL-13, that may contribute toward the underlying inflammation in asthma. In contrast, studies focusing on the role of CD8+ T cells in asthma have been comparatively scarce. Furthermore, most of the data were obtained in murine models, and contradictory results have been produced by various groups, in terms of a possible role for these cells. In this context, possible pro-inflammatory, disease-inducing roles versus protective or “regulatory” roles have been suggested by different studies, as recently reviewed by Baraldo et al. (3). It should be borne in mind that, in the various existing studies, CD8+ T cells were retrieved and characterized using different methodological approaches in terms of patient groups or murine models, biological samples, and study methods. These aspects may account for most of the discrepancies observed, which have indeed been clustering around a non-relevant role (4), a protective role (5), or a disease-favoring role, including association with poorer lung function (6–10) and possibly involving a significant direct and indirect contribution toward a Th2-high bronchial inflammation (11, 12). Nevertheless, it should also considered that different conditions of the local milieu in the target organ, possibly involving exposure to different cytokines and/or antigen-producing cells may also explain discrepant results across existing studies. In addition, some experiments in murine models have also shown that the timing of the experimental setup with CD8+ T cells may also be very relevant in terms of the results observed. In this context, CD8+ T cells that produce high levels of IFN-gamma (Tc1 cells) have been shown to be associated with an attenuation of pulmonary allergic inflammation in rodent models. However, their role appears to depend upon their temporal relationship with the progression of allergic sensitization. In fact, depletion of CD8+ T cells prior to systemic OVA sensitization, either by blocking antibody directed against CD8+ or by using knockout mice, tends to be associated with attenuated allergic inflammation and bronchial hyperresponsiveness (BHR) in response to antigen challenge. However, administration of CD8+ blocking antibody after the initial allergen sensitization procedure results in further increase in BHR and eosinophilia (13).

Finally, it should be emphasized that many studies focusing on CD8+ cells in asthma have utilized different approaches to phenotypically characterizing these cells. In fact, in some cases, it is not clear whether the observed cells are classical CD8+ T cells, regulatory T cells, or NK-like CD8+ T cells.

## General Aspects about the Role of Human NK-Like CD8+ T Cells in Asthma

**Figure 1:**
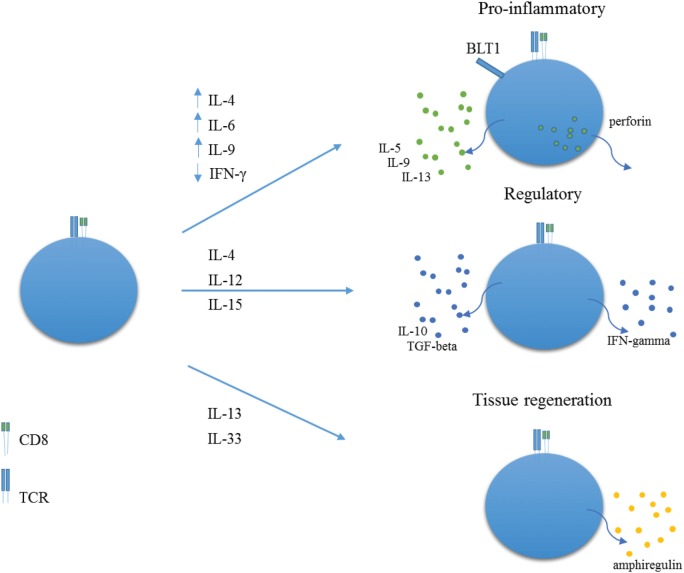
**Proposed roles for NK-like CD8+ T cells**. In environments rich in IL-4, IL-6, IL-9, and low IFN-gamma, NK-like CD8+ T cells may acquire a pro-inflammatory role with expression of BLT1 and production of perforin, IL-5, IL-9, and IL-13. In environments rich in IL-4 and IL-12 or IL-15, NK-like CD8+ T cells may differentiate into regulatory T cells, with production of IFN-gamma, TGF-beta, and IL-10. In pro-inflammatory contexts, rich in IL-18 and IL-33, NK-like CD8+ T cells may produce amphiregulin, thereby promoting tissue regeneration.

As far as we know, there are no published studies on human NK-like CD8+ T cells, as usually defined, in asthma. Human NK-like CD8+ T cells most likely comprise a whole array of cells with different phenotypes and functions, and further research is warranted in order to thoroughly clarify their ontogeny, patterns of differentiation, different phenotypes, and functions (Figure 1). In humans, NK-like T cells are a subset of CD8+ T cells that express prototypical NK cell markers, such as CD56, CD161, CD16, CD94, and CD57, with such expression increasing with aging. In most cases, these CD8+ T cells do not express CD28 (14–16). For the sake of clarity, we will restrict our concept of human NK-like CD8+ T cells to CD8+ T lymphocytes that have been termed concurrently or independently CD28−CD8+ T cells, CD27−CD8+ T cells, CD28−CD57+CD8+ T cells, CD8+ effector memory T cells (T_EM_). A few data exist regarding these possible types of NK-like CD8+ T cells in humans, particularly in the setting of respiratory diseases such as asthma. In addition, given the fact that differences are apparent between these cells in mice and humans, we will only resort to information from murine models where strictly necessary.

CD8+CD28− T cells can be regarded as one of the possible subtypes of NK-like CD8+ T cells. There is a progressive oligoclonal accumulation of CD8+CD28− (and CD57+) T cells with natural aging, possibly due to multiple rounds of immune responses to antigenic exposures (17). In fact, chronic, persistent immune stimulation has been shown to be associated with reciprocal loss of CD28 expression and gain of CD57 expression on human, but not murine, CD8+ T cells (18, 19). Thus, most human CD8+CD28− T cells are CD57+, have shortened telomeres, represent late-differentiated T cells, and have been shown to express high levels of granzymes and perforin (20).

Various other phenotypic markers have been studied on CD8+CD28− T cells, namely CD27, and these phenotypes have been associated with different subtypes of memory T cells. It is generally accepted that human classical memory cells that have differentiated into an effector-like type (T_EM_) tend to be CD27− and CD28−, express perforin and granzymes, have moderate cytotoxic capacity but are capable of producing high levels of cytokines, namely IFN-gamma and TNF-alpha (21–23).

### Can Human NK-Like CD8+ T Cells Have a Pro-Inflammatory Role in Asthma?

In a study involving postmortem peribronchial region samples, the percentage of CD8+CD25+ T cells and perforin expression was higher in patients who had died from asthma (AD) than in asthmatic patients who had died of unrelated causes or in individuals who had died without a history of lung diseases (control groups) (6). In addition, the IFN-gamma/IL-4 ratio was lower in the AD than in the control groups.

Another study, involving asthmatic patients who were followed up for 14 years showed that the decline in lung function (FEV_1_), although mild, was clearly correlated with the number of CD8+ T-cells in airway biopsies, not just at baseline but also on follow-up (5). Curiously, a recent study in asthmatic patients demonstrated that the degree of airflow obstruction (FEV_1_ and FEF_25–75_) was correlated not with the total number of CD8+ T cells but rather with the number of CD8+ T-cells in the bronchoalveolar lavage fluid which expressed the high-affinity receptor for leukotriene B4 (BLT1) and produced IL-13 (10). Furthermore, the numbers of these cells also correlated with serum IgE levels and with the airway basement membrane thickness. However, CD8+ T cells were not further phenotyped in these studies, and we cannot know whether they also contained a subpopulation of NK-like CD8+ T cells.

Other studies have shown that CD8+CD28− (CD57+) T cells may be associated with inflammation in asthma, particularly in more severe cases. In this regard, the induced sputum of asthmatic patients was shown to contain more CD8+CD28− (CD57+) T cells than CD8+CD28+ T cells, in contrast to what was observed in healthy controls. Furthermore, these CD8+CD28− T cells were also shown to be more abundant and to contain lower levels of IFN-gamma in severe asthmatics than in mild asthmatics and age-matched healthy controls. Furthermore, CD8+CD28− (CD57+) T cells from severe asthmatics expressed high levels of intracytoplasmic perforin and demonstrated a clearly more potent cytotoxic activity than in CD8+CD28− T cells from healthy controls and mild asthmatics (24). To what extent this increased cytotoxic activity is relevant to inflammation in asthma still needs to be ascertained.

Clearly, further studies are needed, particularly in terms of the cytokines produced by these CD8+CD28− T cells in asthma. A lower production of IFN-gamma in asthma may be relevant in that it may be less effective at counterbalancing Th2-associated cytokines. In this regard, it should be stressed that, just like CD4+ T cells, CD8+ T cells can also be subdivided into T1-type (Tc1) and T2-types (Tc2), on the basis of the cytokines they produce (25, 26). Thus, some CD8+ T cells produce high levels of IL-4 and/or IL-13, possibly under the influence of IL-4 produced by Th2-type CD4+ T cells, as was shown in a murine model (27). The relevance of IL-4 produced by these CD8+ T cells is not clear, but it may contribute toward tissue remodeling (28). This is even more relevant since IL-4 production has been demonstrated in peripheral blood CD8+ T cells from patients with atopic asthma (29). More importantly, IL-13 may also significantly contribute toward bronchial inflammation, airway hyperresponsiveness, mucus hypersecretion, and tissue remodeling as has been shown in murine models and models of human bronchial epithelium (28, 30, 31).

In addition, a subset of effector memory (T_EM_) CD8+ T cells with high levels of IL-6Ra expression in human peripheral blood was reported. IL-6Ralpha^high^ EM CD8+ T cells actively proliferated, survived, and produced high levels of IL-5 and IL-13. Also, patients with asthma had an increased frequency of IL-6Ralph^high^ CD8+ T cells (T_EM_) in peripheral blood compared with healthy control subjects. It is possible that these cells may serve as a pool, which expands with immune stimulation (32).

Finally, CD8+ T cells may also produce other cytokines that are very relevant to the pathophysiology of bronchial asthma, such as IL-9 (Tc9 cells) and IL-17 (Tc17 cells), which may have a role even in Th2-low settings (11, 33). In fact, a recent study showed that the numbers of peripheral blood Tc2 and Tc17 cells were increased in asthmatic versus non-asthmatic individuals (33). Again, production of these cytokines should be analyzed in human NK-like CD8+ T cells.

### Can Human NK-Like CD8+ T Cells Have a Regulatory Function in Asthma?

In the case of CD8+ T cells, two of the possible ways these cells might have a regulatory function in asthma would be *via* high production of IFN-gamma and/or *via* mechanisms generally associated with “regulatory” T cells and involving the production of IL-10 or TGF-beta or direct cell-cell contact-associated suppression.

Increased expression of IFN-gamma producing CD8+ T cells has been demonstrated in subjects with asthma (12, 34), although a decreased expression of IFN-gamma in CD8+ T cells in atopic asthmatic patients has also been described (35) and CD8+ T cells from atopic asthmatic subjects have been shown to contain more IL-4 than those from non-atopic donors (29). In fact, memory CD8+ T cells can be activated in the presence or absence of specific antigen expressed by dendritic cells, in association with the pro-inflammatory cytokines IL-15 and IL-18, to produce IFN-gamma that leads to the suppression of the underlying Th2-driven allergic airway inflammation (36).

In fact, in the presence of IL-4 and IL-12, murine CD8+ T cells have been shown to become CD39+ Foxp3-negative “regulatory” T cells that demonstrate suppressive activity *via* production of IL-10 and contact-dependent mechanisms (5). Furthermore, memory CD8+ T cells present in the airways of mice after an influenza infection have been shown to suppress the development of subsequent Th2-driven allergic inflammation in an IFN-gamma dependent way (37). In addition, the adoptive transfer of IFN-gamma-producing CD8+ T cells directly into the airways suppressed the allergic response in pre-sensitized mice (36). However, to what extent these CD8+ Tregs are CD28− has not been described.

It is thought that naïve CD8+CD25+ cells can differentiate into CD8+ Tregs in the presence of antigen and the relevant cytokines (38). As an example, human CD8+ Treg can be generated in the presence of IL-4 and IL-12; these cells are CD25+Foxp3+ and are capable of secreting IL-10, TNF-alpha, IFN-gamma as well as granzymes (39). Furthermore, these cells have been shown to block the activation of naïve or effector T cells, to suppress IgG/IgE antibody responses (39), IL-4 expression, and the proliferation of CD4+ T cells (40). However, most of these cells described in humans are CD28+ (39–41) and most likely do not involve NK-like CD8+ T cells. An alternative pathway in terms of CD8+ T cell differentiation toward Tregs may involve IL-15. In this context, human CD8+CD56− T cells, stimulated with IL-15, were shown to acquire the capacity to secrete IFN-gamma, IL-1beta, TGF-beta, and IL-10, suggesting a regulatory phenotype (42).

It should be stressed that a subset of human CD8+CD28− T suppressor cells, which were shown to act upon antigen-presenting cells, rendering them tolerogenic to CD4+ T cells were described in a model of mixed lymphocyte reaction (43). Phenotypic analyses of these CD8+CD28− T cells showed that they were CD3+, CD5^high^, CD8^high^, CD27+, CD56−, CD62L+ (44) opening up the possibility of the existence of CD8+CD28− Tregs in humans.

In human asthma, flow cytometry analysis showed an increased percentage of CD8+CD28− T cells in peripheral blood of adult allergic asthmatics compared to controls (45). In addition, patients with severe asthma had a higher percentage of CD8+CD28− and CD8+CD28−TCRalpha/beta+CD62L^high^ FoxP3^bright^ T cells than the other groups after enrichment, suggesting that these cells might not be immunosuppressive or that their increased numbers in asthma might indicate a tissue damage-limiting function, as happens in the context of viral infections [reviewed by Josefowicz et al. (46)]. In contrast, the same group of researchers showed that the percentages of peripheral blood CD8+CD25+FoxP3^bright^ T cells of patients with severe asthma or mild to moderate asthma were markedly lower than those of non-asthmatic controls (47). Curiously, the percentages of CD8+CD25+FoxP3^bright^ T cells correlated with mean peak expiratory flow (PEF%) values in these asthmatic patients (47). Although this study did not analyze whether these CD8+ Tregs were CD28− (and/or CD57+), joint analysis of the results from the studies by these researchers may suggest that the CD8+CD28− described in their reports are not true immunosuppressive Tregs, which is in line with results from various other groups that have described CD8+CD28− T cells as essentially cytotoxic and not immunosuppressive (24, 48–51). Furthermore, other authors have also shown that human CD8+CD57+ T cells are mostly cytotoxic, at least those that are present in the context of autoimmune diseases (52–56).

Nevertheless, the picture is not clear at all, since a clear immunosuppressive activity carried out by CD8+CD57+ T cells (57–59) as well as by CD8+CD28− T cells (44, 60–63) has been described, but in the context of tissue transplantation and autoimmune diseases in humans. To what extent this may apply to allergy and asthma needs to be determined.

Thus, in order to clarify this issue of human NK-like (CD8+CD28−) T cells having or not “regulatory” properties in human asthma, further studies are needed, involving a thorough phenotypic and functional characterization of both peripheral blood and bronchial CD8+CD28−CD57+ as well as CD8+CD28−CD57− T cells in patients with bronchial asthma of different degrees of severity as well as in non-asthmatic controls.

### Can Human NK-Like CD8+ T Cells Have a Tissue-Regenerating Function in Asthma?

Amphiregulin is an epidermal growth factor ligand, which apparently promotes tissue repair under inflammatory conditions [reviewed by Berasain and Avila (64)]. Studies in a murine model have shown that, through the production of amphiregulin, Treg cells have a direct role in lung tissue repair and maintenance during viral infection that is independent of their suppressive activity and is induced by different stimuli (65). In this context, the pro-inflammatory cytokine IL-18 and the alarmin IL-33, which are upregulated in the context of inflammation and tissue damage, induced *in vitro* amphiregulin production by Treg, independently of TCR stimulation. However, this study did not fully clarify which type of Tregs was involved, although mostly CD4+ Tregs were studied.

Amphiregulin is also produced by human T cells, as shown in a study in which signaling through the TCR induced amphiregulin expression by most or all human T cell subsets in peripheral blood, including naive and memory CD4+ and CD8+ T cells, Th1 and Th2 *in vitro* T cell lines, and subsets of memory CD4+ T cells (66). In these different T cell types, amphiregulin synthesis was regulated essentially by acute signals, which may be appropriate for tissue repair. Importantly, amphiregulin-producing CD4+Foxp3+ Tregs have been described in damaged tissues, namely muscles in murine models (67). Furthermore, the specialized proresolving mediator maresin-1 has been shown to reduce asthma-associated bronchial inflammation in a murine model, and this was associated with an increased expression of amphiregulin and *de novo* generation of CD4+ Tregs (68). It is, thus, possible that damaged bronchial tissue in cases of severe asthma may be associated with the local accumulation of amphiregulin-producing Treg. However, amphiregulin expression has not been studied in human NK-like, CD8+CD28− T cells. In addition, amphiregulin expression in asthma must be interpreted with caution, since elevated levels of amphiregulin have been detected in induced sputum in asthmatic children, where its levels correlated with the numbers of sputum eosinophils and sputum eosinophil cationic protein (69). Furthermore, there was a significant negative correlation between sputum amphiregulin and lung function (FEV_1_). Finally, another study in asthmatic children showed that levels of amphiregulin in the sputum were increased in disease exacerbations (47). In addition, in this study, amphiregulin was also shown to induce proliferation of normal human bronchial epithelial cells, which may be associated with tissue remodeling in asthma.

## Conclusion

Independently of other reasons, seemingly contradictory findings regarding human CD8+ T cells, namely NK-like CD8+ T cells may be due to different populations/subpopulations involved in the different experimental setups used. In addition, a clearer definition of the CD8+ subsets both in phenotypic and functional terms would allow more useful comparisons between studies both in animal models (rodent) and in human pathological contexts.

## Author Contributions

Literature search and writing up of mini-review: OL, AF, and LT-B.

## Conflict of Interest Statement

The authors declare that the research was conducted in the absence of any commercial or financial relationships that could be construed as a potential conflict of interest.
